# Effect of metastatic site on survival in patients with neuroendocrine neoplasms (NENs). An analysis of SEER data from 2010 to 2014

**DOI:** 10.1186/s12902-020-0525-6

**Published:** 2020-04-03

**Authors:** Nikolaos A. Trikalinos, Benjamin R. Tan, Manik Amin, Jingxia Liu, Ramaswamy Govindan, Daniel Morgensztern

**Affiliations:** 1 Department of Medicine, Washington University in St. Louis, St. Louis, USA; 2 Division of Oncology, Washington University in St Louis, 660 S. Euclid Avenue, Campus Box 8056-29, St Louis, MO 63110 USA; 3 Department of Surgery, Washington University in St. Louis, St. Louis, USA

**Keywords:** Neuroendocrine tumors, Metastasis, SEER database, Survival

## Abstract

**Background:**

Neuroendocrine neoplasms (NENs) display variable behaviors based on origin and grade. We assumed that both tumor origin and the location of metastasis may play a role in survival.

**Methods:**

We queried the SEER database (2010–2014) for patients with an established diagnosis of NENs and documented site of metastasis and identified 2005 patients. Overall survival (OS) at the time points were estimated by the Kaplan-Meier method Cox proportional-hazards models were used to evaluate the relationship of the interested variables and OS.

**Results:**

Lung, liver, bone and brain metastases were observed in 9, 77, 7 and 6% of metastatic patients respectively. In the multivariate model, metastasis locations were significantly associated with worse survival (liver HR: 1.677 (1.226–2.294); (bone metastasis HR: 1.412 (0.965–2.065); brain HR: 1.666 (1.177–2.357)). We produced a scoring system based on site of origin, metastasis location, age, gender, histology and tumor size that can stratify metastatic NEN patients in low, intermediate and high-risk categories to help physicians with decision making.

**Conclusion:**

Site of metastasis plays an important role in survival of metastatic NEN patients independent of commonly described prognostic factors and should be considered in survival estimates.

## Background

Neuroendocrine neoplasms (NENs) are rare malignancies of the aerodigestive, genitourinary and integumentary systems. Their histologies vary from well-to-moderately differentiated neuroendocrine tumors (NETs) to poorly differentiated neuroendocrine carcinomas (NECs) and their natural history has been described in several publications [1–3]. Most studies are limited due to the small number of cases, inconsistent follow-up or retrospective nature but it is clear, however, that the incidence of NENs is increasing [3] and that, at least for certain subtypes, survival might be improving [2].

NENs show a spectrum of behaviors and this makes their treatment challenging. Some exhibit an indolent, slow growth pattern, while others parallel the more aggressive, rapidly spreading tumors such as small cell lung cancer (SCLC); in between there are neoplasms of intermediate malignant potential. Research so far has identified stage, site of origin [4] and differentiation [5 6], as well as proliferative indices (Ki-67, mitotic count) as important prognostic factors and multiple scores have been published, trying to predict survival in metastatic disease or recurrence after curative surgery [5–11]. In general, well- differentiated tumors progress slowly and surveillance may be the best approach in some cases, whereas poorly differentiated neoplasms require urgent aggressive chemotherapy and are associated with markedly shorter survivals [12]. Tumors of small bowel origin tend to have a better prognosis [13] compared to NENs originating in the pancreas. The effect of other factors such as age, race [3], resectability [14], performance status [15] or even marital status [16] has similarly been examined in several publications. Most medical decisions nowadays consider tumor of origin, staging, but also tumor differentiation and mitotic indices (values that have formed the basis of the current grading system [17]).

While it is generally accepted that stage IV (presence of metastasis) portends a poor prognosis for most neoplasms including NENs, there is no consensus on the gravity and importance of metastatic sites, or how they interplay with the primary tumor site when it comes to survival estimates. The National Cancer Institute’s Surveillance, Epidemiology, and End Results (SEER) Program is an annually updated population data bank which has been used extensively to monitor long term trends in survival trends for rare and common tumors alike. The November 2017 iteration of the program covers 28% of the US population and includes 10,050,814 cases. A variety of publications have utilized the power of SEER data to draw conclusions about NENs [18, 19]. It has been shown for example that the incidence of certain types of NETs has increased and that the survival of patients has improved over time [2]. This has been partially attributed to treatments such as somatostatin analogues [20] (time to progression prolonged by 8 months), targeted therapies such as everolimus [21] and sunitinib [22] (progression free survival benefit of about 5 months for both), and hopefully pazopanib or peptide receptor radionuclide therapy (PRRT) [23] in the future. With the latest iteration, the SEER database was enriched to include details of general metastatic sites, including lung, liver, bone and brain. This presents a unique opportunity to study the behavior of metastatic neuroendocrine tumors across a range of sites and histologies. We sought to explore the behavior of NENs with regards to the site of origin and metastatic areas and hypothesized that site of metastasis will carry different prognostic significance depending on tumor grade and tissue of origin.

## Methods

### Data source

We queried the SEER database on the November 2017 submission. We specified the time frame from 2010 to 2014. We identified NENs by ICD-O3 histology codes based on prior relevant publications [3] and the following diagnoses: Carcinoid tumor (8240), enterochromaffin cell carcinoid (8241), neuroendocrine carcinoma (8246), atypical carcinoid tumor (8249), malignant enterochromaffin-like cell tumor (8242), large cell neuroendocrine carcinoma (8013), mixed pancreatic malignant endocrine and exocrine tumor (8154), insulinoma (8151), glucagonoma (8152), malignant pancreatic endocrine tumor (8150), gastrinoma (8153), somatostatinoma (8156), vipoma (8155). We excluded any diagnosis code related to small cell lung cancer, mixed adenoneuroendocrine carcinoma and goblet cell carcinoid. We extracted the following variables: Age; sex; marital status; histology; grading; origin; site of metastasis and survival. As this is publicly available, de-identified data, no institutional review board (IRB) approval was required.

### NEN classification

We relied on SEER histologic grade information to classify cases as Grade 1 /G1 or well differentiated; G2 or moderately differentiated; G3 or poorly differentiated; and G4 or undifferentiated / anaplastic. Grade 3 and 4 tumors have similar survival characteristics as per prior publications [3], are being treated with the same regimens and thus we grouped them together in the high grade category. For our analyses we only included patients with a single general site of metastasis (liver, lung, bone or brain) and where the grade was documented. We codified tumors of origin into the following 4 groups: Small bowel, lung, pancreatic and other/miscellaneous. Lung NENs are generally classified into typical and atypical carcinoids, large cell and small cell neuroendocrine carcinomas but for this analysis we relied on histology and grade documentation and the small cell subgroup was excluded.

### Statistical analysis

The clinical characteristics were summarized using descriptive statistics. Kaplan-Meier (KM) curves for OS were generated that provide unadjusted survival estimates for the patients and across strata. Differences between strata were determined by log-rank tests. Cox proportional-hazards models were used to evaluate the relationship of the interested variables and OS. The proportionality assumption was tested by adding a time-dependent covariate for each variable. The variables with *p* < 0.20 from univariate models are considered in the multivariate model. The final multivariable model was built using the backward stepwise selection approach to identify all significant risk factors. Factors significant at a 10% level were kept in the final model. A prognostic score was developed by assigning hazard ratios to each variable in the final multivariable model, All statistical tests were two-sided using an α = 0.05 level of significance. SAS Version 9.4 (Cary, NC) was used to perform all statistical analyses.

## Results

### Patient characteristics

In total, out of 31,650 NEN patients we identified 2005 patients with adequate grading information and a single documented site of metastasis (liver, lung, bone or brain), spanning all tumor types diagnosed between 2010 and 2014. Details are presented in Table 1. Median age was 63 years (13–95) and 52% of patients were male. The majority of patients were white (81%), married (58%) and insured (96%). The primary site of origin was lung in 22% pancreatic in 23% and small bowel in 27% of cases. About 28% of NENs originated in other areas and were designated “other”, details are included in the Appendix 1 and Appendix 2. Well and moderately differentiated tumors comprised about 61% of cases and 39% were atypical/high grade. About 9% had metastasis to the lung, 77% to the liver, 7% to the bone and 6% to the brain with no site overlap.

**Table 1:** Patient characteristics. CL: Common law marriage, SNF: Skilled Nursing Facility, DP: Domestic Partner. Other: Please refer to Appendix

Parameter	N	Percent
Age group		
**< 60**	781	38.95
**60–70**	684	34.11
**> 70**	540	26.93
Sex		
**Male**	1038	51.77
**Female**	967	48.23
Race		
**White**	1636	81.6
**Black**	264	13.17
**Other**	96	4.79
**Unknown**	9	0.45
Marital status		
**Married / CL**	1163	58
**Unmarried or DP**	5	0.25
**Widowed**	198	9.88
**Divorced**	226	11.27
**Single**	307	15.31
**Unknown**	106	5.29
Insurance		
**Insured**	1398	69.73
**Medicaid**	228	11.37
**Nonspecific**	294	14.66
**Uninsured**	57	2.84
**Unknown**	28	1.4
Location of diagnosis		
**Hospital inpatient/ outpatient/ surgery**	1976	98.55
**Private physician**	22	1.1
**Laboratory**	6	0.3
**SNF/hospice**	1	0.05
Primary site		
**Lung**	437	21.8
**Other**	569	28.38
**Pancreas**	453	22.59
**Small Bowel**	546	27.23
Tumor size		
**< =35**	832	52.89
**> 35**	741	47.11
**Frequency Missing = 432**		
Histology		
**Typical**	496	24.74
**Atypical/ high grade**	1487	74.16
**Pancreatic**	22	1.1
Grade		
**Well differentiated or Grade 1**	850	42.39
**Moderately differentiated or Grade 2**	382	19.05
**Poorly differentiated or Grade 3**	564	28.13
**Undifferentiated / anaplastic**	209	10.42
T status		
**N/A**	422	21.05
**T1 (all versions)**	119	5.94
**T2 (all versions)**	340	16.96
**T3 (all versions)**	649	32.37
**T4 (all versions)**	468	23.34
**T0**	7	0.35
N status		
**Node negative or microscopic**	598	29.83
**Node positive**	1148	57.26
**N/A**	259	12.92
M status		
**Metastatic**	1891	94.31
**N/A**	114	5.69
Tumor size		
**< =35**	832	52.89
**> 35**	741	47.11
**Frequency Missing = 483**		
Liver metastasis		
**Yes**	1553	77.46
**No**	437	21.8
**N/A**	15	0.75
Lung metastasis		
**Yes**	186	9.28
**No**	1750	87.28
**N/A**	69	3.44
Bone metastasis		
**Yes**	142	7.08
**No**	1808	90.17
**N/A**	55	2.74
Brain metastasis		
**Yes**	124	6.18
**No**	1826	91.07
**N/A**	55	2.74
Surgery		
**Performed**	954	47.58
**Not advised**	1013	50.52
**Advised, not performed**	27	1.35
**N/A**	11	0.55

#### Survival by tumor of origin, grade and location of metastasis

**Fig. 1:**
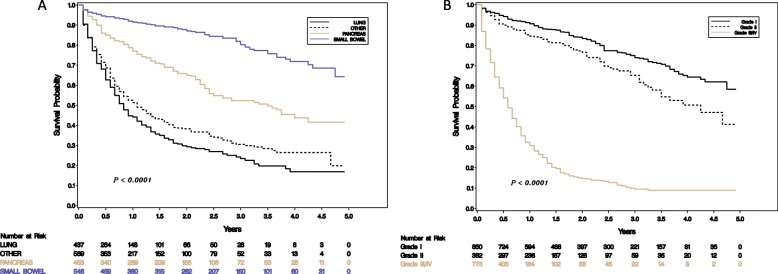
Survival according to tumor site of origin (**a**) and grade (**b**)

**Fig. 2:**
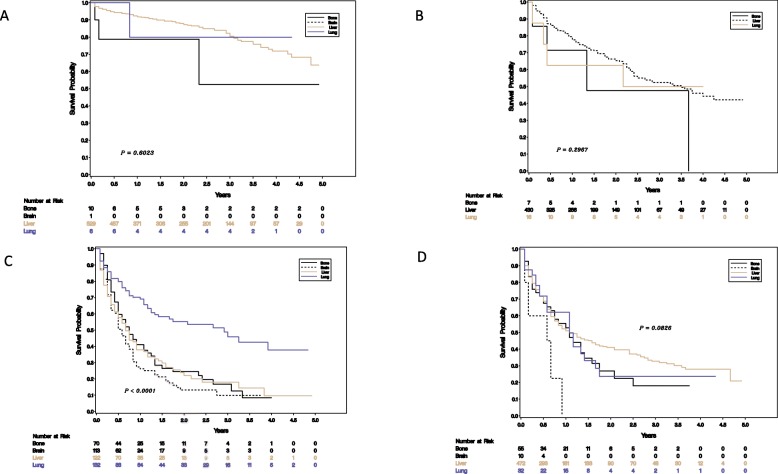
Survivals for tumors originating in the bowel (**a**), pancreas (**b**), lung (**c)** and other (**d**) by site of metastasis (bone, brain, liver lung)

The median OS for metastatic lung NENs as a whole was 0.83 years, for pancreas it was 3.5 years, for “other” was 1.08 years and it has not been reached for small bowel origin (Appendix 2). Poorly differentiated tumors had a median overall survival of 0.58 years, for grade 2 it was 4.25 years and not reached for grade 1 tumors. Patients with lung origin or higher grades tended to have poorer survivals (Fig. 1) and for patients with lung origin, location of metastasis had a significant effect on survival (Fig. 2).

### Univariate and multivariate OS analysis

Univariate analysis (Table 2) showed the prognostic significance of age, race, sex, marital status, insurance coverage, site of origin, histologic grade and tumor size at the level of 0.05. Setting of initial diagnosis and presence or absence of lymph node involvement were not significant. The final multivariate model included age at diagnosis (< 60, 60–70, > 70), sex, grade (1, 2, 3&4, insurance status, primary site and site of metastasis and tumor size (<=35, > 35) (Table 3). It showed that the metastasis locations were significantly associated with the worse survival ((liver HR: 1.677 (1.226–2.294), bone HR: 1.412 (0.965–2.065), brain HR: 1.666 (1.177–2.357)). Same applied to primary sites including lung (HR: 2.901 (2.027–4.15)), other (HR: 2.541 (1.836–3.516)), and pancreas (HR: 1.502 (1.091–2.069)) which had worse survival compared to bowel site. Hazard ratios for high grade histology were predictably worse (HR: 2.062 (1.525–2.787)) and this also applied to original tumors more than 35 mm (HR: 1.274 (1.065–1.525)).

**Table 2:** Univariate analysis for Overall Survival (OS)

Parameter	***P*** value	Hazard Ratio	95% CI	
**Age**	<.0001	1.03	1.024	1.036
**60–70 vs. < 60**	<.0001	1.553	1.312	1.839
**> 70 vs. < 60**		2.363	1.996	2.797
**Male vs nonmale**		1.266	1.106	1.45
**White vs. other**	0.0006	0.951	0.692	1.309
**Black vs. other**	0.7932	1.014	0.708	1.451
**Non-married vs. Married**		1.202	1.045	1.382
**Uninsured vs. insured**	0.0099	1.252	0.853	1.837
**Hospital vs. non-hospital**	0.2514	1.105	0.61	2.003
**Lung vs. Bowel**	0.7421	6.869	5.39	8.754
**Other vs. Bowel**	<.0001	5.659	4.458	7.182
**Pancreas vs. Bowel**	<.0001	2.692	2.074	3.494
**Atypical vs typical**	<.0001	4.487	3.507	5.742
**Pancreatic vs typical carcinoid**	<.0001	2.178	1.047	4.53
**Grade 2 vs. Grade 1**		1.49	1.16	1.913
**Grade 3/4 vs. Grade 1**	<.0001	8.544	7.155	10.202
**Node positive vs. node negative**	0.1727	1.116	0.953	1.307
**Tumor size**	<.0001	1.012	1.01	1.014
**Tumor size > 35 vs. <=35**	<.0001	2.287	1.942	2.694

**Table 3:** Multivariate analysis for Overall Survival (OS) and prognostic score

Parameter	***P*** value	Hazard Ratio	95% CI		Score
**Original Site**					
**Lung**	<.0001	2.901	2.027	4.15	2.9
**Other**		2.541	1.836	3.516	2.5
**Pancreas**		1.502	1.091	2.069	1.5
**Bowel**		1			1
**Metastasis location**					
**Bone**	0.0066	1.412	0.965	2.065	1.4
**Brain**		1.666	1.177	2.357	1.7
**Liver**		1.677	1.226	2.294	1.7
**Lung**		1			1
**Grade**					
**1**	<.0001	1			1
**2**		1.348	1.011	1.797	1.3
**3&4**		4.534	3.545	5.799	4.5
**Age**					
**< 60**	<.0001	1			1
**60–70**		1.257	1.028	1.537	1.3
**> 70**		2.062	1.687	2.519	2.1
**Gender**					
**Male**	0.0007	1.328	1.127	1.565	1.3
**Female**		1			1
**Histology**					
**High grade /atypical**	<.0001	2.062	1.525	2.787	2.1
**Pancreatic**		1.894	0.881	4.071	1.9
**Typical**		1			1
**Tumor size**					
**> 35**	0.0082	1.274	1.065	1.525	1.3
**< =35**		1			1
**Score group**	**Frequency**	**Percent**		**Cumulative** **frequency**	**Cumulative** **percent**
**Low: < 9.5**	516	32.8		516	32.8
**Medium: 9.5 -** **13**	537	34.14		1053	64.94
**High: >13**	520	33.06		1573	100
**Frequency Missing = 432**					

Total score is the scores sum of non-missing variables. Its minimum and maximum are 7 and 15.9, respectively

### Survival score

We created a scoring system based on the results of the multivariate survival analysis to assign survival categories in patients with metastatic NENs. Details are shown in Table 3. Both site of origin and site of metastasis were significantly and independently associated with survival outcomes, with lung origin and brain/liver metastasis portending the poorest prognosis. Other significant factors from the model have been previously described and include age > 70, grade, sex and tumor size. We assigned score values to every factor and established three score thresholds: 7–9.5, 9.5–13 and 13–15.9 corresponding to a low, intermediate and high-risk category. The score is calculated by adding the individual scores of age, sex, tumor size, insurance status, grade, site of origin and metastatic location.

## Discussion

Neuroendocrine neoplasms are rare and heterogeneous, but their incidence is rising, their evolution can span multiple years [1 5] and new treatments have been approved in the past decade. In order to have meaningful discussions about prognosis and properly design clinical trials, clinicians need to better understand the factors affecting patient survival. In this paper we have used the SEER database to examine metastatic only patients and showed that both the site of origin and the metastatic site independently influences their survival. We have further attempted to quantify that and provide a prognostic score for use by the clinician in everyday practice.

The varied behavior of patients based on tumor histology and grade is not new knowledge. Multiple publications have shown that, on average, patients with small bowel neuroendocrine tumors fare better compared to pancreatic neuroendocrine tumors (PanNENs) and that higher grade is associated with decreased survival. Yao et al. [3] examined more than 35,000 SEER NEN cases from 1973 to 2004 and showed inferior survival for patients with pancreatic or lung NENs compared to those of the small intestine, something that we have also shown here. More recent publications have shown slightly improved but similar trends between histologies [24] and better survival. Similar to our findings, poorly differentiated tumors had lower median survivals compared to the well differentiated counterparts, reflecting the virulent biology and the limited advances in the treatment of high grade NENs in the past 20 years. Our study adds to this knowledge with detailed estimates for tumors of varied origins and metastatic sites, as well as with a prognostic score for better stratification of the patients. This score emphasizes the importance of metastatic location and shows that brain and liver involvement carry a higher risk of death regardless of other well-known factors, such as origin of the tumor or histology.

It is important to mention that we relied on a uniform reporting style for all origins, including lung neuroendocrine tumors. This has some inherent problems, as classification for lung NENs is different (typical and atypical carcinoids, small and large cell neuroendocrine categories). The WHO classification that designated the typical and atypical categories was developed for resected primary carcinoids, so its usefulness in the metastatic setting is not clear. Moreover, small cell lung cancer, with its well described histology and extensive treatment options, was removed from our database. Our approach in this paper is similar to other large SEER studies [3 24] but can definitely alter the reported results. The GI NEN literature is moving to a more uniform grading of neoplasms and there is a trend to use proliferation markers to predict outcomes in lung NENs as well [25, 26] but this is far from a settled argument. Another issue is that classification of pancreatic NENs (and most recently other GI NENs) has changed to include a category of “well differentiated, high grade” with a prognosis intermediate between G2 and G3. We have approached this problem by factoring in our model both typical/atypical/pancreatic histology and G1/G2/G3 categories but acknowledge that it can cause problems with interpretation of data.

Our results are consistent with the published literature. A study in two major institutions [27] in carcinoid patients with bone and/or liver metastases confirmed that coexistence of bone and liver metastasis was indicative of worse prognosis with a significant difference in overall survival - the small total numbers of patients (691) might have made it more difficult to obtain statistical significance in other histologies. The poor prognosis of brain metastases is not unexpected and is consistent across different tumor types, such as breast [28, 29], lung [30], gastrointestinal [31] and ovarian [32]. It is usually measured in matters of months and may reflect the virulence of the tumor, the severity of CNS dysfunction or the inability of most current therapies to cross the blood brain barrier. In our study, median survival was a mere 7 months and it is worth mentioning that single site brain metastases were more prominent in lung and “other” primary but uncommon in pancreas and small bowel (Appendix 2) and carried the same poor prognosis. Although not analyzed in this study, the median survival of patients with multiple metastatic sites (594 cases identified in our cohort) was expectedly diminished with a median OS of 6 months (95% CI: 6–6.96). The rate of skeletal metastases was relatively low at 7% (compared with 10% in published literature) and conversely, the rate of brain metastasis was high (6%, compared with 0.5% in non SCLC NENs and about 20% in SCLC) [33]. One needs to remember that this is an enriched database, consisting of only M1 patients with single site metastasis and with exclusion of SCLC. In prior iterations of this study, the database included 34,704 unselected patients with any metastatic entry (M0/M1) and there the metastatic sites included brain (2.99%), bone (4.63%), liver (14.82%) and lung (4.10%); still higher, but closer to, actual reported registry data.

The effect of tumor of origin in patients with similar metastases is a poorly understood phenomenon and can be approached by the significantly different genetic makeup of these neoplasias. Genetic syndromes such as multiple endocrine neoplasia type 1 (MEN1), Von Hippel-Lindau (VHL) and neurofibromatosis type 1 (NF1) have been associated with NENs, but they only account for about 10% of observed cases. PanNENs have demonstrated a variety of alterations [34] including inactivation of TSC1/2 and ATRX/DAXX genes and involve alterations in DNA damage repair, chromatin remodeling, mTOR signaling and telomere maintenance [35]. In contrast, whole-exome sequencing on small intestinal NENs (SI-NENs) has shown pretty low mutation rates [36] and it is felt that epigenetic processes such as DNA methylation or histone modifications might be more important in tumor propagation and metastasis. Pulmonary carcinoids share a lower rate of mutations compared to their adenocarcinoma counterparts (including very low rates of TP53 and RB1 gene mutations), but have frequent mutations in chromatin-remodeling genes [37, 38]. High grade NECs on the other hand demonstrate a very aggressive behavior and poor prognosis with higher frequencies of TP53 mutations [39] and RB1 alterations [40], albeit lower than those reported for small cell lung cancer (SCLS). While the above cannot explain, they can definitely suggest different behaviors of various NETs in the metastatic state, in the sense that lower mutation burden/higher reliance on epigenetic processes can be associated with a more indolent behavior.

Our analysis has several weaknesses. It relies on retrospective information collected over a limited period of time. The patient sample was ultimately limited to less than 2200 cases, as we insisted on full sets of data that included grade, origin and single metastatic site. While SEER will document metastasis, only 4 major sites were codified (lung, liver, bone and brain) and there is no data on how that was established. About 13% of patients had no known N status, which, in the setting of metastasis, is of little importance. Moreover, there is concern about accurate reporting of M stage metastasis itself in SEER; some pathologic M0 patients are known to have clinical M1 status. We tried to address this issue by including only patients with a documented site of metastasis; we can assume that most of these have been clinical. The adoption of receptor imaging techniques such as radiolabeled octreotide or Gallium Dotatate PET/CT scans, which can identify occult metastatic disease is not uniform or consistent between practices. This can underestimate the true incidence of the metastatic population in SEER data. Reporting of histology and grade has changed over time, especially for PanNENs and estimation of Ki-67 is notoriously difficult [41], therefore some patients might have been misregistered. We were unable to calculate median survivals for some groups, as the data (especially for small bowel tumors) had not had time to mature. For the majority of patients, we can assume that metastases were not confirmed by biopsy, and we were unable to distinguish between oligometastatic and heavy tumor burden disease. There was no information on grade switch or transformation to more aggressive tumors. Surgery was not included in our analysis, as it was incompletely reported and it is still debatable if optimal cytoreduction plays a role in survival [42]. The data includes no information on initial or subsequent treatments, which can significantly affect survival; for example, everolimus and sunitinib were approved between 2010 and 2014. Our numbers were not large enough to further subcategorize metastatic NENs and allow for multiple comparisons; this will potentially be feasible in subsequent SEER iterations. Finally, our score reflects the results of the analysis and can help guide prognosis estimation but has not been validated in a separate dataset of patients (plans to perform it in an institutional database currently underway). Future studies should incorporate the effect of tumor mutations, especially in low and intermediate grade NENs and allow for comparisons between different treatments.

## Conclusion

Site of metastasis plays an important role for survival in metastatic NEN patients and is probably reflective of variable tumor biology, even among NENs of similar origin and grade. It is independent of commonly described prognostic factors and should be considered in survival estimates and design of clinical trials.

## Data Availability

We used the SEER*Stat software, available online from https://seer.cancer.gov/data-software/, in the client-server mode. The datasets obtained and analyzed in this study are available in the SEER database, https://seer.cancer.gov/data/.

## Appendix 1

**Table 4:** A Other site clarification

Subsite	N	Percent
**Esophagus**	31	5.45
**Stomach**	79	13.88
**Other GI (large bowel, rectal, anal)**	313	55.01
**Hepatobiliary**	20	3.51
**Pelvic/ peritoneal**	7	1.23
**Bladder**	12	2.11
**Renal/ureters**	23	4.04
**Prostate**	13	2.28
**Uterus and ovaries/vulva**	14	2.46
**Skin**	4	0.7
**Adrenal glands**	1	0.18
**Breast**	5	0.88
**Non lung mediastinal**	47	8.26
**Frequency Missing = 1436**		

## Appendix 2

**Table 5:** B Median survivals by tumor and metastatic site in years

Tumor of origin	Median	M-lung	M-liver	M-bone	M-brain
Lung	0.83	2.92	0.67	0.75	0.58
Pancreas	3.5	N/A	3.5	1.33	N/A
Small bowel	N/A	N/A	N/A	N/A	N/A
Other	1.08	1.08	1.08	1.08	0.58

## Abbreviations

CI: Confidence interval
G1(2,3): Grade 1(2,3)
GI-NEN: Gastrointestinal neuroendocrine neoplasm
HR: Hazard ratio
IRB: Institutional review board
KM: Kaplan meier
MEN1: Multiple endocrine neoplasia type 1
NEC: Neuroendocrine carcinoma
NEN: Neuroendocrine neoplasm
NF1: Neurofibromatosis type 1
OS: Overall survival
Pan-NEN: Pancreatic neuroendocrine neoplasm
PRRT: Peptide receptor radionuclide therapy
SCLC: Small cell lung cancer
SEER: Surveillance, epidemiology and end results program
SI-NEN: Small intestinal neuroendocrine neoplasm
VHL: Von Hippel-Lindau
WHO: World health organization
